# The Scalp as a Donor Site in Pediatric Burns: Systematic Review of the Literature and Proposal of a Management Algorithm

**DOI:** 10.3390/ebj7020024

**Published:** 2026-05-08

**Authors:** Carlotta Paola Maria Canonica, Irene Paraboschi, Eleonora Durante, Francesca Izzo, Anna Mandelli, Sara Costanzo, Elvira Conti, Gloria Pelizzo, Anne Le Touze

**Affiliations:** 1 Pediatric Surgery Department, Buzzi Children’s Hospital, 20154 Milan, Italy; 2 Pediatric, Urologic, Plastic Surgery and Pediatric Burn Unit, Clocheville Hospital, Centre Hospitalier Régional Universitaire (CHRU Tours), 37000 Tours, France; 3 Medicine and Surgery Faculty, University of Milan, 20122 Milan, Italy; 4 Department of Biomedical and Clinical Science, University of Milan, 20157 Milan, Italy; 5 Anaesthesia and Intensive Care, Buzzi Children’s Hospital, 20154 Milan, Italy; 6 Plastic and Reconstructive Surgery, Armand-Trousseau Hospital, Assistance Publique Hôpitaux de Paris (APHP), 75012 Paris, France

**Keywords:** pediatric burns, scalp, STSG, management algorithm

## Abstract

**Highlights:**

**What are the main findings?**

- Scalp is an optimal donor site, and it is widely used in pediatric burns.
- Evidence points out scalp as safe and effective for STSG in pediatric patients.

**What are the implications of the main findings?**

- A management algorithm improves clinical practice and elevates standard of care.

**Abstract:**

Background: Deep burns in pediatric population often require split-thickness skin grafts (STSGs) and the identification of an optimal donor site is crucial to minimize morbidity, accelerate healing and reduce short- and long-term complications. The scalp appears to be increasingly used in clinical practice, but evidence remains limited, despite the promise of novel bioengineering and regenerative approaches. Methods: A systematic review about the use of scalp for STSG in pediatrics was conducted across PubMed, Scopus, and Cochrane (2005–2025). Clinical outcomes considered were donor-site healing time, early and late complications, perioperative practices, and quality of scars. Results: Four studies met the inclusion criteria (*n* = 417, mean age 2.9–7.3 years) with follow-up periods up to 27 years. Epithelialization occurred between 7 and 25 days. Early complications included folliculitis (up to 44% in certain hair types) and delayed healing (*n* = 13; 52%). A rigorous harvesting technique is needed to avoid preventable complications. Late sequelae included alopecia (1.6% to 33%—the latter largely unperceived by patients) and hypertrophic scarring (1.6–4%). Scar quality was rated good in >80% of cases. Conclusions: Evidence supports the scalp as a safe, efficient, and cosmetically favorable donor site for pediatric STSG. Based on evidence and clinical experience, we propose the first structured scalp-donor management algorithm to optimize safety, reduce complications, and standardize perioperative care in the management of pediatric burns.

## 1. Introduction

Burn injuries in the pediatric population represent a complex condition that requires specialized management in an environment adapted to the specific needs of children [1]. They constitute a true therapeutic challenge due to unique physiological characteristics of the pediatric population, their long-term impact on growth and development, and the significant psychological burden they impose [2,3].

Management of pediatric burn patients additionally requires a specialized approach aimed at reducing stress and pain, considering the often-prolonged hospitalization and the high level of expertise required in reconstructive surgery [4,5,6].

Currently, surgical treatment of burns involves early excision and grafting—ideally within the first days after injury—which reduce the risk of sepsis, hypertrophic scarring, and contractures and are associated with improved survival and shorter hospital stays in extensively burned children [7,8,9,10].

**Figure 1:**
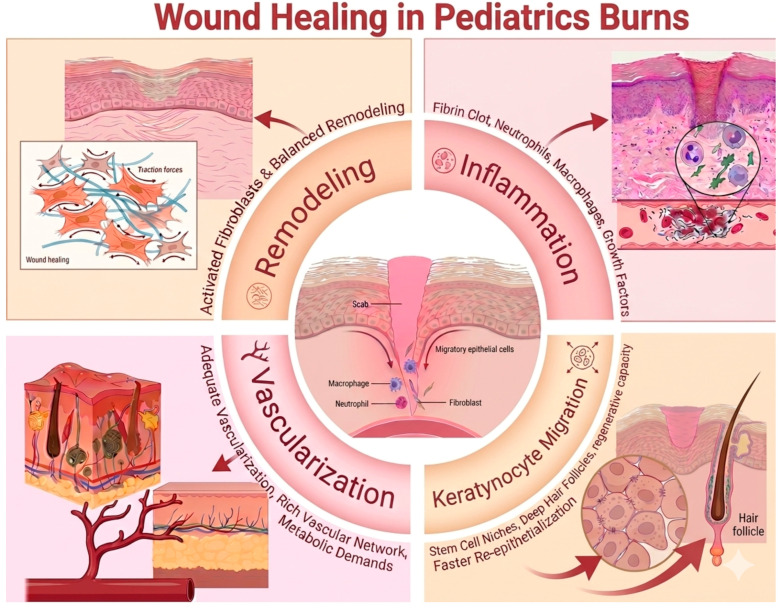
Wound healing mechanism in pediatric burns [21].

Wound healing in pediatric burn patients is a dynamic and tightly regulated process driven by a complex cascade of cellular and molecular events [11]. Understanding the effectiveness of the scalp as a donor site requires a detailed examination of the mechanisms underlying tissue regeneration (Figure 1). Immediately following graft harvesting or tissue injury, a fibrin clot forms, providing a provisional scaffold for the recruitment of inflammatory cells such as neutrophils and macrophages [11]. These cells play a crucial role in wound debridement and in the release of key growth factors, including TGF-β, which initiate and regulate subsequent healing phases [12]. A major advantage of the scalp as a donor site lies in its unique regenerative capacity. Keratinocyte migration occurs not only from the wound edges but predominantly from epithelial stem cell niches located within deep hair follicles and sebaceous glands. The high density of these adnexal structures in the pediatric scalp contributes to significantly faster re-epithelialization compared to other anatomical regions [12,13]. Consequently, preservation of the deeper dermal layers during harvesting is essential to maintain this regenerative potential. Adequate vascularization is another critical factor, as it supports the high metabolic demands of tissue repair in children [14]. The scalp dermis is characterized by a rich vascular network, which further enhances healing efficiency [15,16]. In parallel, activated fibroblasts migrate into the wound bed; in pediatric patients, this response is particularly robust and must be carefully modulated to prevent excessive extracellular matrix deposition and the development of hypertrophic scarring [17,18]. Following the initial reparative phase, collagen remodeling and scar maturation continue over several months. The use of the scalp as a donor site promotes more balanced and harmonious tissue remodeling, reducing the risk of dyschromia and contractures, which are more commonly observed in areas exposed to greater mechanical tension.

One of the main challenges in pediatric burn care is identifying an adequate donor site for skin grafting: in extensive burns, it is frequently difficult to find a large enough area with suitable characteristics (homogeneous pigmentation, hair density, skin thickness) [7]. The success of this process depends not only on the management of the recipient site but also on the judicious selection of an appropriate donor site. Conventional donor areas, such as the thighs, buttocks, and trunk, may be insufficient in children, particularly in cases of extensive burns or in patients who require repeated grafting [16,19,20].

Over the past decades, the use of the scalp as a donor site for autologous skin grafts in children has gained increasing acceptance, as identifying a suitable donor area is often complex in the pediatric population [16,22,23].

While novel technologies such as 3D bioprinting [24] and bioengineered autologous dermo-epidermal skin grafts [25] are setting new directions in regenerative medicine, their application in pediatrics is often constrained by research limitations. Consequently, scalp harvesting remains one of the mainstay techniques for achieving STSG in pediatric patients.

Despite these advantages, clinical protocols for scalp harvesting remain heterogeneous, and the literature consists of only a small number of studies, each using different techniques, dressings, and postoperative regimens. As a result, a need to consolidate existing evidence and to propose a unified management approach emerges as a priority.

The absence of shared guidelines restricts the harmonization of international protocols, limiting the overall effectiveness of pediatric burn care. The resulting challenges are twofold: the scarcity of pediatric-specific data and the unique characteristics of severe burn injuries that complicate the establishment of standardized—especially surgical—approaches.

The purpose of this paper is to systematically review the currently available literature on the use of the scalp as a donor site for split-thickness skin graft (STSG) in children and to formulate a structured management algorithm to standardize its clinical use.

By synthesizing findings from a systematic literature review, this study proposes a management algorithm designed to facilitate clinical decision making and standardize protocols, ultimately ensuring superior outcomes in pediatric burn care.

## 2. Materials and Methods

**Figure 2:**
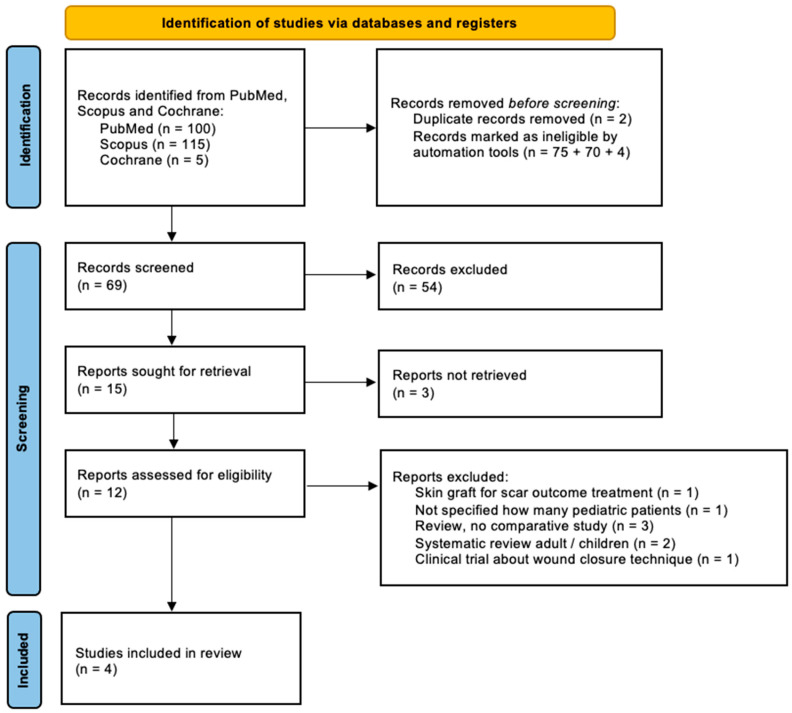
PRISMA flowchart of study selection.

A systematic review of the literature was conducted across PubMed, Scopus and Cochrane Library databases from the past twenty years (2005–2025) to identify studies addressing the use of the scalp as a donor site for STSG in pediatric patients, with the objective of assessing clinical applications, risks, and benefits. Studies published within the past 20 years were included to provide an updated perspective on the use of the scalp in pediatric burn management. Despite its established role in clinical practice, the available evidence remains limited, underscoring the need for broader consensus and more robust data to support standardization. Articles were selected if language of publication was English and included meta-analyses, clinical trials, original research articles and reviews; case reports were excluded. The search strategy, detailed in the PRISMA flow diagram (Figure 2), used combinations of terms related to the scalp, donor sites, skin harvest techniques, and burns. The protocol for this systematic review has been archived and is accessible in the Open Science Framework (OSF) (DOI: 10.17605/OSF.IO/HCEPD); PRISMA 2020 checklist is available at Table S1. Eligible studies focused on pediatric populations (mean age: 5.7 years) undergoing scalp harvesting for STSG in the context of burn care. Studies were required to report at least one clinical outcome relating to donor-site healing, complications, dermatome settings, or overall postoperative course. Reviews, adult-only studies, or studies lacking extractable data were excluded. Data extraction centered on patient demographics, number of harvests, operative technique, depth parameters of the dermatome, perioperative management, postoperative dressing, epithelialization time, early and late complications, and, when available, scar quality.

## 3. Results

### 3.1. Results

The process followed a PRISMA approach beginning with the identification of 220 records, which were subsequently screened through titles and abstracts. After removing duplicates and excluding studies that did not meet the criteria, only four studies fulfilled the requirements for inclusion (Figure 2).

The four studies included a total of 417 children, with ages ranging from 4 months to 15 years. Follow-up periods varied widely, extending from approximately 18 months to more than 27 years. Despite the heterogeneity in sample size and methodology, several consistent findings emerged across all studies. A summary of the included studies is shown in Table 1.

In two studies most of the population was composed of Caucasians, while, in Van Niekerk et al.’s study [23], the majority was represented by Black African descent children. In three studies (Rotatori et al. [26], Neuhaus et al. [22], Van Niekerk et al. [23]) TBSA% mean was >20% (respectively 26%; 25.59%; 44.92%) and most of the accidents were caused by scald or flame (respectively 25.7% and 61.6%; 53.1% and 40.6%; 24% and 76%).

All reports described the exclusive use of electric dermatomes, with harvesting depths typically ranging from 0.15 mm to 0.30 mm depending on the child’s age and the surgeon’s preference.

Preparation of the scalp consistently involved antiseptic cleansing prior to harvesting and adrenaline-soaked compresses afterward to control bleeding and injection of diluted adrenaline solutions into the subgaleal plane to facilitate hemostasis and uniform depth during harvesting. Some teams also used lubricating agents to improve the smoothness of dermatome passage.

Regarding postoperative management, dressings varied among centers but commonly included calcium alginate sheets, Bactigras^®^, Xeroform^®^, Omniderm^®^, or transparent film dressings. Many clinicians left the dressing untouched until spontaneous detachment to avoid damaging newly regenerating epithelium, while others performed dressing changes every 48 h, depending on institutional preference.

Healing occurred remarkably quickly. In one series from Wyrzykowski et al. [15], complete epithelialization of the donor site was achieved between the 7th and 10th postoperative day, particularly in younger children. In one large series from Van Niekerk et al. [23], a median time of 25 days was reported; however, this included a heterogeneous population with differing hair characteristics. Ethnic variability was evident, particularly in children with hair types VI–VII (following De La Mettrie classification [27]), predominantly Black African descent patients, who demonstrated slower donor-site healing, occasionally extending beyond two weeks [23].

Early complications were infrequent and generally mild. Folliculitis was the most common issue, particularly in children with tightly curled hair, and, in the cohorts described by Van Niekerk et al. [23], reached up to 44%; in contrast, no early complications were described by Neuhaus et al. [22] and Wyrzykowski et al. [15]. Delayed healing was also predominantly observed in these patients, with a percentage reported by Van Niekerk et al. of 52% [23]. Beyond these issues, early infection and significant bleeding were exceptionally rare.

**Table 1:** Studies included in the systematic review.

Author (Year)	No. of Patients	Mean Age (yrs)	Follow-Up Period (yrs)	Harvests (No. of Patients)	Type and Depth Setting of Dermatome (cm)	Perioperative Management	Postoperative Management	Epithelization Time (Days)	Early Complications (n/% Total)	Late Complications (n/% Total)	Quality of Scars (Evaluation Scale If Used)
Rotatori et al. (2019) [26]	237	7 ± 5	2.4	44 (Scalp)	Electric dermatome	Cleaning with chlorhexidine—infiltration RL + epinephrine 1:100,000/L prior to harvesting—epinephrine-soaked pads after harvesting	Calcium alginate dressing/transparent film dressing/silver-impregnated dressing	21 (median)	0/0	Alopecia (1/2), HTS (1/2)	P–HTS at any time (*p* < 0.0001)
Neuhaus et al. (2019) [22]	32	7.3 ± 4.5 (1–15 yrs)	27.09 ± 3.04 (13–31)	Single and multiple	Electric dermatome, 0.15–0.3 mm	Hairline marked, shaved completely and washed with water and soap + antiseptic—physiological saline subgaleal injection prior to harvesting—epinephrine-soaked pads after harvesting	Single-layer gauze removed after overnight moisturizing	n.d.	0/0	Alopecia (11/33.38)	No HTS, no patients aware of alopecia areas
Van Niekerk et al. (2018) [23]	25	5.7 (4 months–12 yrs)	1.59	Single (5)–multiple (20)	Electric dermatome, 0.2 mm	Hairline marked, shaved, chlorhexidine wash + hypochlorite solution—application of warm adrenaline (1:30,000) solution and subdermal injection of solution of bupivacaine 0.01% and adrenalin 1:500,000 mixed with 0.9% saline	Antiseptic dressing (Omniderm^®^/Xeroform^®^/Bactigras^®^)	25 (median: 15.1 Black Africans; 11.8 mixed race; 8.5 Caucasians)	Folliculitis (11/44) hair type VI–VII, Black African descent; non-healing wounds (13/52)	Alopecia (4/16), HTS (1/4)	n.d.
Wyrzykowski et al. (2015) [15]	123	2.98 (4 months–15 yrs)	≥10	Single (121)–multiple (2)	Electric dermatome, 0.2 mm < 7 yrs–0.3 mm > 7 yrs	Shaved—application of solution of disinfectant—subgaleal injection of adrenalin solution (1:250,000–500,000)—application of adrenaline-soaked gauzes	Antiseptic dressing (Bactigras^®^)	Between 7 and 10	0/0	Alopecia (2/1.6), forehead scar (1/0.8), HTS (1/0.8)	Good/very good 80.9%—satisfactory 16.2%—unsatisfactory 2.9% (Vancouver Scar Scale)

Abbreviations: yrs = years; RL = Ringer Lactate; HTS = hypertrophic scar.

Late complications were similarly limited. Alopecia occurred in a small minority of patients, typically ranging from 1.6% to 4% in most series (Rotatori et al. [26] reported alopecia rates of 2%, similarly to Wyrzykowski et al. that reported 1.6% [15]; Van Niekerk et al. [23] reported a rate of alopecia of 16% and notably all the patients that presented this complication were of Black African descent or mixed race. One study from Neuhaus et al. [22] reported a higher rate of alopecia up to 33%; however, the authors noted that the affected patients at long-distance follow-up (up to 30 years) rarely perceived the alopecic areas, suggesting that, even when present, the cosmetic impact may be negligible.

Hypertrophic scarring (HTS) was reported in 1.6 to 4% of children, in particular one large series from Wyrzykowski et al. [15] found a rate of HTS of 1.6% and systematically evaluated scar quality using the Vancouver Scar Scale, finding that more than 80% of patients exhibited good or very good outcomes. Similarly, Rotatori et al. [26] found an HTS rate of 2% and Van Niekerk et al. a rate of 4% [23]. Forehead scarring, attributed to dermatome friction or imprecise edges, was exceedingly rare (0.8% of cases in Wyrzykowski et al.’s series [15]). Long-term complications were infrequent even after reharvesting the scalp and were all related to ethnicity—as reported by Van Niekerk et al. [23], all seven patients who presented with long-term complications were Black African or mixed race with respectively hair types VI–VIII or III–V [27]. Notably, these complications occurred after the first or second harvest, none after the third procedure.

Taken together, the findings across all four studies strongly supported the scalp as a highly favorable donor site in pediatric burn reconstruction. Its exceptionally rapid healing, low complication profile, and hidden cosmetic impact placed it at an advantage over traditional donor areas.

### 3.2. Proposed Management Algorithm

**Figure 3:**
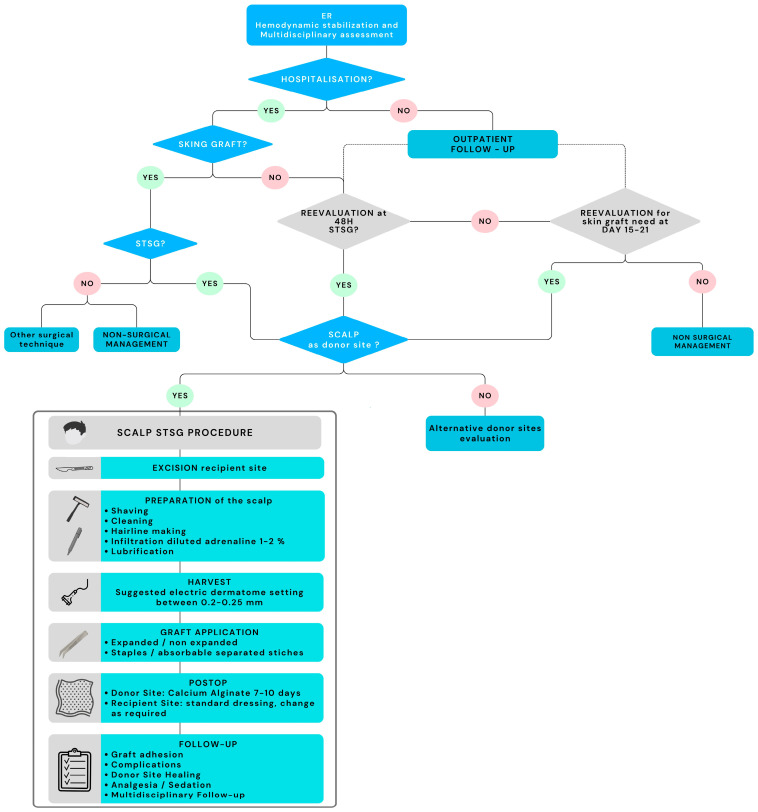
Proposed Management Algorithm. Abbrev. ER = Emergency Room; STSG = split-thickness skin grafting; POSTOP = postoperative period.

Based on the synthesis of the literature and the structured workflow presented in Figure 3, a comprehensive management algorithm has been formulated to optimize management of pediatric burn patients requiring STSG.

This algorithm integrates initial burn assessment, donor-site planning according to patient-specific characteristics, and appropriate surgical techniques to promote healing, minimize complications, and preserve functional and aesthetic outcomes.

Standardizing such an algorithm may be particularly helpful for emerging or developing burn centers, while considering the unique physiological and psychological needs of pediatric patients. Early, standardized care pathways are essential to optimize long-term functional and aesthetic outcomes and to minimize sequelae.

If a split-thickness skin graft (STSG) is deemed an appropriate option, the scalp should be considered as a primary donor site. However, caution is warranted, as the available evidence is based on a limited number of studies. A personalized approach remains essential, particularly in the context of pediatric care. Also, considerations about TBSA to be covered should be individualized and tailored on the child’s need.

The scalp—especially in children under 6 years of age—serves as a highly advantageous donor site because of its rapid healing, low incidence of hypertrophic scarring, and suitability for repeated harvests. The high density of follicles and epidermal stem cells supports robust epithelial regeneration and excellent cosmetic outcomes, even after successive harvests. Proper patient selection and technique are key to minimizing short- and long-term complications such as focal alopecia, chronic folliculitis, and hair transfer to recipient sites [13,15,16,28,29,30].

Donor-site management plays an essential role in overall burn treatment. Studies confirm that scalp harvesting is widely practiced and offers an excellent risk–benefit profile, yielding satisfactory short- and long-term results [15,22,23,26]. Nevertheless, rigorous patient assessment remains crucial to reduce complication risk [23].

Clinical decision making should begin with the evaluation of the child’s overall condition, ensuring hemodynamic stability and identifying indications for skin grafting. The assessment of burn depth and severity guides the decision-making process based on standardized criteria. A surgical indication may be established immediately or within two weeks, especially for mosaic burns or delayed healing. Even in outpatient cases, re-evaluation is essential to assess the possible need for grafting in borderline situations.

Once the indication for STSG is confirmed, the scalp should be considered the donor site of choice, and the clinician must assess the availability of healthy, unburned scalp. The selection of this donor site should also consider the child’s ethnic origin and hair type, as these factors influence healing dynamics and complication rates.

The technique must follow rigorous, standardized protocols to reduce complications and ensure optimal outcomes, as reported in the flowchart in Figure 3 with specific materials and settings. In particular, during scalp preparation, the hairline should be marked to ensure correct harvesting [22,31]. Dermatome depth should be tailored to individual needs, considering an optimal setting of 0.20–0.25 mm and adjusting based on operator technique.

The management algorithm aims to improve quality of care and strengthen team training, emphasizing the need for structured organization and continuous education. It could serve as a decision-support tool, integrating diagnostic and therapeutic pathways.

Standardization of clinical pathways and ongoing training for multidisciplinary teams are essential to improve outcomes. Centralization of pediatric burn care into specialized centers ensures better surgical expertise, superior postoperative supervision, and alignment with evidence-based practices. Long-term follow-up remains indispensable for monitoring functional recovery, scar development, and psychosocial adaptation.

## 4. Discussion

Pediatric burns represent a therapeutical challenge since their pathophysiological features distinguish their management from those of adults, primarily due to differences in body composition, immune response, and developmental stage [2,32]. A tailored strategy is necessary to minimize stress and pain, in particular considering pediatric population characteristics and needs [4,5,6]. Children have a higher surface-area-to-body-mass ratio, which increases evaporative losses and predisposes them to hypovolemic shock, heightened metabolic demands, susceptibility to infection, and delayed recovery when compared to adults [32].

Furthermore, as growth and development are ongoing, burn injuries may disrupt normal physical and psychological maturation, with scars and contractures affecting mobility and growth [9]. These specificities necessitate a pediatric multidisciplinary approach and long-term follow-up to manage the acute and chronic sequelae inherent to pediatric burn injuries [4,5,6]. From a surgical point of view, STSG is preferred for extensive burns due to its ability to cover large areas and the possibility of reusing the same donor site after healing [13]. Common donor sites include the thigh, buttocks, and, in children, the scalp [15,16]. The literature consistently highlights that the scalp represents a valuable alternative to other donor sites, allowing minimization of scarring while ensuring successful graft take [15,16,23,26,28,33,34]. The scalp is especially advantageous due to rapid re-epithelialization, low risk of hypertrophic scarring, possibility of frequent reuse, and minimal postoperative pain due to its immobility [15,16,28,29]. All the studies reviewed collectively suggest that the scalp is a safe and effective donor site for pediatric STSG [15,22,23,26].

Young patients, particularly in cases of scald burns, frequently necessitate grafting due to the thinness of pediatric skin and smaller body surface [1,13,35,36,37,38].

Technically, dermatome settings between 0.15 and 0.3 mm appear optimal, and rapid re-epithelialization is consistently reported. Ethnicity and hair type significantly influence healing times and folliculitis risk. Indeed, longer healing time was reported by Van Niekerk et al. [23] for children of Black African descent (median 15.1 days) compared to Caucasian patients (median 8.1 days) and folliculitis was described in 44% of patients of Black African descent.

Long-term complications—mainly alopecia and hypertrophic scarring—were uncommon and generally mild. The hidden nature of scalp scarring promotes high patient satisfaction, as demonstrated by the fact that even with a meticulous analysis of the scalp minimal areas of alopecia were reported, of which the patients were not aware [22].

The primary reasons supporting the use of the scalp as a donor site for STSG in burned children include its rapid re-epithelialization—typically within 5 to 10 days—which reduces infection risk and allows early reharvesting if needed, and its low incidence of hypertrophic or keloid scarring, even after multiple procedures. This advantage derives from the high density of hair follicles and epidermal stem cells, facilitating robust regeneration and producing superior cosmetic outcomes [15,16,28,29,30].

The scalp can therefore be used repeatedly at short intervals, making it particularly valuable for children with extensive burns and limited availability of conventional donor sites [15,16,28]. The ability of the scalp to be reharvested at short intervals makes it more useful rather than the absolute area obtained per session. In a large series of 450 pediatric burn patients with mean TBSA of 46%, the scalp was harvested a mean of 2.2 times (range 1–10) with a mean interval of 6 days between harvests [16].

The literature reviewed here described cases in which children underwent repeated scalp harvesting with minimal cumulative morbidity, an option that is rarely feasible with other donor sites. Early complications were rare, although folliculitis was reported by Van Niekerk et al. at a high rate (44%), notably related to hair type [23]. Late complications included alopecia and HTS (with mean percentages respectively of 13% and 2.5%). Indeed, compared with conventional donor sites such as the thighs, buttocks, or trunk, the scalp consistently demonstrates superior outcomes in terms of healing time, postoperative discomfort, and cosmetic sequelae [15,16]. Traditional donor areas in children are more prone to hypertrophic scarring and hyperpigmentation, particularly in certain skin phototypes [19,26]. They are also associated with more intense postoperative pain, which can complicate early mobilization, hygiene, and psychological comfort. In contrast, the scalp is less exposed to friction, rarely undergoes hypertrophic responses, and permits early return to normal activities [15,16,28,29].

Advances in regenerative medicine and emerging technologies are progressively reshaping the landscape of skin reconstruction and may further refine harvesting strategies in the future [24,25,39]. Among advanced regenerative strategies, 3D bioprinting has emerged as a sophisticated approach to fabricate multilayered skin substitutes by precisely arranging living cells and biomaterials to mimic native tissue architecture. However, while this technology offers a promising future for burn care, it remains largely in the preclinical phase and lacks specific clinical validation in pediatric cohorts [24,40,41,42]. In this context, the following table (Table 2) provides a comparative overview of the scalp as a donor site alongside alternative skin regeneration approaches, including dermal substitutes and 3D bioprinting. Although these innovative techniques show considerable promise, the scalp continues to represent a highly effective and reliable option in pediatric patients, owing to its rapid healing capacity, low morbidity, and immediate availability.

Nevertheless, a rigorous harvesting technique and strict exclusion of previously burned or scarred scalp areas are essential, as these constitute major risk factors for chronic folliculitis or alopecia [16,23]. Furthermore, in children of Black African descent (hair types VI–VIII), the risk of delayed healing, folliculitis, and alopecia is higher, making the scalp a less favorable donor site in this population [23].

In summary, evidence from the literature, confirmed by data extracted from this systematic review, demonstrates that donor-site selection plays a central role in the management of pediatric burn patients requiring autologous skin grafting. The scalp, owing to its rapid regenerative capacity, low risk of hypertrophic scarring, and suitability for repeated harvests, represents a valuable alternative to classical donor sites such as the thigh or back.

Currently, no standardized algorithm exists in the medical literature specifically addressing the use of the scalp as a donor site for STSG in pediatric burns, including detailed management of complications such as alopecia, chronic folliculitis, non-healing donor wounds, and hair transfer to recipient sites [15,16,28,29,30]. Available studies emphasize the importance of meticulous technique, avoidance of previously burned areas, and careful patient selection to minimize adverse outcomes [16,23,42].

The reviewed studies also displayed methodological heterogeneity, as all were retrospective and single center. Differences in postoperative dressing protocols, harvesting depths, and assessment of complications prevent the formation of high-level evidence. Also, limitations to the algorithm’s wide application include the expertise required for scalp harvesting—an advanced technique demanding extensive training—and the dependence on adequate institutional infrastructure (multidisciplinary burn centers, pediatric-trained teams, specialized anesthesia, rehabilitation services). The studies included in this review were restricted to pediatric populations, which inherently limited the overall sample size and reduced the generalizability of the findings. Consequently, the available evidence reflects highly specific and context-dependent observations, underscoring the need for larger, more diverse cohorts to strengthen the robustness and external validity of the conclusions. These limitations make the development of a standardized management algorithm particularly valuable, as it can offer clinicians a unified approach based on the best available data and practical experience as well as standardized procedures and safer outcomes for children.

**Table 2:** Comparison between skin grafting techniques in pediatric burn management.

Strategy	Mechanism/Technology	Advantages	Disadvantages	Results/Effectiveness	References
Scalp (STSG)	Autologous split-thickness skin graft from the cranial vault.	High stem cell density, rapid re-epithelialization (7–10 days), inconspicuous scar.	Risk of alopecia, folliculitis (in specific hair types), procedural pain.	Excellent. Gold standard for large pediatric burns; allows multiple harvests.	[15,16,22,26,30]
Traditional STSG (Thigh/Back)	Autologous graft from trunk or limbs.	Large surface area available.	Visible scarring (donor site morbidity), slower healing, higher pain scores.	Good, but limited by aesthetic concerns and donor-site exhaustion.	[19,38]
3D Bioprinting Implants	Layer-by-layer deposition of cells (bioink) and scaffolds.	Patient-specific geometry, no donor site morbidity.	High cost, complex regulatory approval, lack full vascularization.	Experimental. Promising for future use but currently lack long-term clinical data.	[24,39,40,41]
Dermal Substitutes (e.g., Integra, Suprathel)	Scaffold-based regeneration (bovine collagen/GAG).	Improved elasticity, useful for full-thickness burns.	Require two-stage procedure, high risk of infection, high cost.	Very effective when combined with ultra-thin STSGs.	[10,43,44]
Cultured Epithelial Autografts (CEAs)	In vitro expansion of patient’s own keratinocytes.	Minimal donor tissue needed to cover massive areas.	Fragile, long preparation time (3 weeks), high cost, poor mechanical strength.	Variable. Reserved for massive burns (>50% TBSA).	[45,46]

## 5. Conclusions

Skin grafting in children, in particular STSG, remains the technique of choice in pediatric burn care, when applied with a targeted, minimally invasive strategy, optimizing the balance between efficacy and safety. Although a limited number of studies were included in the review, considering exclusively a pediatric population, evidence shows that the scalp emerges as an effective and safe donor site, offering rapid healing, low morbidity, and excellent cosmetic results, while allowing repeated harvesting in cases of extensive burns in pediatric patients.

Recently, regenerative medicine has offered new perspectives for pediatric burn treatment, but the scalp remains the gold standard, providing superior biological healing and immediate clinical reliability compared to currently available advanced technologies.

The proposed management algorithm provides a structured, evidence-based framework for patient selection, operative technique, and postoperative care. Its adoption by experienced multidisciplinary teams may reduce complications, shorten hospital stays, and improve functional and aesthetic outcomes in children undergoing skin grafting for burns.

## Data Availability

The original contributions presented in this study are included in the article. Further inquiries can be directed to the corresponding author.

## Supplementary Materials

The following supporting information can be downloaded at: https://www.mdpi.com/article/10.3390/ebj7020024/s1, Table S1: PRISMA 2020 checklist [47].

## Abbreviations

The following abbreviations are used in this manuscript:

STSG	Split-thickness skin graft
HTS	Hypertrophic scarring
